# Saving lives by asking questions: nurses’ experiences of suicide risk assessment in telephone counselling in primary health care

**DOI:** 10.1017/S146342362200055X

**Published:** 2022-10-26

**Authors:** Rikard Wärdig, Ann-Sofie Engström, Annelie Carlsson, Frida Wärdig, Sally Hultsjö

**Affiliations:** 1 Department of Health, Medicine and Caring Sciences. Division of Nursing and Reproductive Health, Linköping University, Linköping, Sweden; 2 Department of Psychiatry, Ryhov County Hospital, Jönköping, Sweden; 3 Department of Health, Medicine and Caring Sciences, Division of Nursing and Reproductive Health, Linköping University, Linköping, Sweden; 4 Division of Primary Health Care, Region Östergötland, Linköping, Sweden

**Keywords:** conventional content analysis, nurse, primary care, suicide risk assessments, Sweden, telenursing

## Abstract

**Aim:**

To explore nurses’ experiences of suicide risk assessment in telephone counselling (TC) in primary health care (PHC).

**Background:**

Globally, priority is given to developing suicide prevention work in PHC. However, suicide risk assessments in TC are not included in these interventions even though these are a common duty of nurses in PHC. More expertise in the field can contribute to knowledge important for developing nurses’ tasks within PHC.

**Methods:**

A qualitative interview study was conducted with 15 nurses. Data were analysed using conventional content analysis.

**Findings:**

As suicide risk assessment in TC is a common duty for nurses in PHC, they need to be listened to and given the right conditions to perform this work. The nurses lack training in how to carry out suicide risk assessments and are forced to learn through experience. Intuition guides them in their work. A prerequisite for making correct assessments over the telephone is that the nurses are given time as well as the right competence. The PHC organisation needs to create these conditions. Furthermore, interventions to support suicide prevention need to include strategies to help nurses perform suicide assessment in TC.

## Introduction

Suicide is a major public health concern and a common cause of death (McDowell *et al*., 2011). Globally, it is estimated that 700 000 people die from suicide every year (World Health Organization (WHO) 2022a). Suicide is thus one of the leading causes of death in the world, and in 2020, approximately 1500 died through suicide in Sweden (The Swedish Public Health Agency, 2021). Nearly 90% of suicides are related to mental disorders (Bachmann, 2018). The burden of mental disorders continues to grow with significant consequences for public health in all countries (WHO 2022b). WHO strives to raise awareness of suicide and to make suicide prevention a high priority globally (WHO 2022a). Despite this, only a few countries have included suicide prevention among their health priorities. Health care professionals in primary health care (PHC) have the important task of making risk assessments as they represent the first-line care and have an extensive contact area with the population (Cross *et al*., 2019). PHC has a whole-of-society approach to health that aims at ensuring the highest possible level of health and well-being and their equitable distribution by focusing on people’s needs, as early as possible along the continuum from health promotion and disease prevention as close as feasible to people’s everyday environment (SFS^2017^:30; WHO & UNICEF 2018; WHO 2022c). Several studies show that significant numbers of people, approximately 40%, who commit suicide have visited PHC in the month prior to their suicide, and 80% in 1 year prior to their suicide (Leavey *et al*., 2017; Hauge *et al*., 2018; Cross *et al*., 2019; Stene-Larsen and Reneflot, 2019; Harmer *et al*., 2021). Of these, females, and persons ≤50 years are most represented (Stene-Larsen and Reneflot, 2019), as females are more likely than men to use PHC services (Gonzalez *et al*., 2018). Making suicide risk assessments is complex as the patient’s actions can be erratic and some patients do not affirm suicidal thoughts (Oquendo and Bernanke, 2017). It is always important that health care professionals ask questions about thoughts and plans to commit suicide when needed (McDowell *et al*., 2011) because this can otherwise be missed and lead to incorrect treatment. The WHO describes patient safety as an intervention to reduce the risks of the patient being injured in care (WHO 2021). The prerequisites for patient safety are that the patient is involved, that care is promoted by a good safety culture in which staff have adequate competence and there is clear management (National Board of Health and Welfare 2021b).

Many of the contacts with PHC take place via telephone counselling (TC), which is why its quality is of great importance for patient safety (Röing *et al*., 2013). Nevertheless, difficulties arise in dealing with suicidal patients in PHC due to a lack of education (Leavey *et al*., 2017) and knowledge of how to make correct suicide risk assessments (Runeson *et al*., 2017). A clear structure is especially important in TC because it is easy to make mistakes as it is not possible to see and assess the patient’s facial and bodily expressions (Khan, 2013). Inadequate communication in TC can lead to misunderstandings and delayed treatments (Ernesäter *et al*., 2009). Nurses face several challenges in TC. Lack of time can lead to the conversations ending prematurely and thus being of poor quality, which can eventually lead to an incorrect assessment. Decision support systems can facilitate nurses’ work and provide security (Ernesäter *et al*., 2009; Purc-Stephenson and Thrasher, 2010). However, these systems can be perceived as incomplete, controlling and as contrary to the nurses’ own opinions. Nurses are an important part of suicide risk assessment in TC, because they are the ones who mainly talk to patients by phone. Studying this area can lead to increased knowledge of the challenges that nurses face and may be helpful in the development of future PHC.

Thus the aim of this study was to explore nurses’ experiences of suicide risk assessment in TC in PHC.

## Methods

### Study design and sample

As the field has been sparsely explored, a descriptive qualitative design was considered appropriate as this design aims to illuminate and describe events in daily life as experienced and described by participants themselves (Sandelowski, 2000). Conventional content analysis is a suitable method for providing comprehensible and detailed illustrations of different experiences of the same phenomenon (Hsieh and Shannon, 2005).

### Participants and setting

A purposeful sample was used (Polit and Beck, 2021). To ensure that all participants had experiences of suicide risk assessment in TC, nurses were recruited to the study from PHC centres where TC is a recurrent part of the nurse’s work. A total of 15 nurses participated in the study, of which all were women. Eight of the nurses were registered nurses and seven were district nurses. They varied in age between 30 and 66 years (average age, 45 years) and their experience of working in TC in PHC varied between 2 and 25 years (average age, 9 years).

### Data collection

Head managers of nine different PHC centres in southern Sweden were contacted and received information about the study. After their approval, the head managers were asked to forward information about the study to nurses who had experience of making suicide risk assessments in TC. If the nurses were willing to participate, contact information was forwarded to the authors who then contacted them. To achieve a calm and relaxed atmosphere during the interview, the nurses could select the place for the interview (Patton, 2015), of which 10 were performed in secluded rooms at the nurses’ workplaces and 5 were performed digitally via zoom due to the Covid-19 pandemic. Before the interviews, the nurses received information about the purpose and implementation of the study and written informed consent was obtained. Those interviewed through zoom sent their written informed consent by post to the interviewers.

### Interviews

In conventional content analyses, the proposed method of collecting data is semi-structured interviews (Hsieh and Shannon, 2005). The interviews were conducted between October 2021 and December 2021 individually by RW, AC and AE. A semi-structured interview guide with open-ended questions was created to capture descriptive stories (Hsieh and Shannon, 2005). Initially, the interview began by allowing the nurses to talk freely about their experiences of working with TC, and then in a more structured way following an interview guide produced by all the authors. The interview guide consisted of the questions: *What are your experiences of making suicide risk assessments in TC*? *How do you experience these conversations? What is the reason you carry out suicide risk assessments?* To achieve greater depth in the interviews, probing questions were asked, for instance about the challenges they experienced in relation to the phenomenon studied.

To encourage the participants to give answers that were rich in content, they were asked to describe specific situations and conversations in which suicide risk assessments were carried out and what was needed for these conversations to be further developed. Three test interviews were conducted, as no corrections were made in the guide, and these were included in the study. The interviews lasted between 18 and 49 min, with median 33 min. After each interview, there was time for reflections between informants and interviewers to make sure nobody would leave the room before they were ready. Further notes were written down to capture the researcher’s thoughts about what had happened or emerged during the interview. All interviews were recorded digitally and then transcribed verbatim.

### Data analysis

The analysis followed conventional content analysis, in accordance with Hsieh and Shannon (2005). After the interviews had been transcribed, the texts were read carefully several times by all the authors to create an understanding of the whole. Then parts of the texts that seemed to capture important concepts to answer the aim were marked (meaning-units). Longer statements were shortened to find the most central part of each answer. During this phase, notes were kept about impressions and thoughts that arose to facilitate the further analysis. Codes were sorted together based on their content in a primary coding scheme consisting of 13 primary subcategories. The subcategories were then compared with each other and those with similar content were divided into four categories. Finally, a tree diagram was created where the categories were assigned a hierarchical structure where one category appeared to be superior to the rest. During the whole analysis, participants’ own words were used to avoid as much interpretation as possible. Quotations from raw data were identified for the purpose of ensuring trustworthiness of the content of the categories when presenting the results (Hsieh and Shannon, 2005). The analysis went back and forth in a dynamic process between the data as a whole and the different parts of the analysis (Polit and Beck, 2021). Example of meaning units, codes, sub category and category are presented in Table 1.

**Table 1.** Example of meaning units, codes, sub-category and category

Meaning units	Codes	Sub-category	Category
*They may already feel down and then you might destroy their self-confidence even more by insulting them to ask - Do you want to take your own life? But at the same time, I think you save lives by asking anyway*	Asking for suicidality	**When patients seek help for mental illness**	**REASONS FOR SUICIDE RISK ASSESSMENTS**
*Then I ask a straightforward and honest question… Have you ever considered harming yourself? Then, you can open up possibilities for more such questions. I think it’s easier if I ask that question than if they themselves express it (P3).”*	Asking for risk of self-harming behaviour	**When patients seek help for mental illness**	

### Ethical considerations

The study was approved by the Ethics Committee of Linköping, Sweden (Dnr 2016/343-31). Written informed consent was obtained from the participants in accordance with the Declaration of Helsinki (WMA, 2013) to protect the participants. The researchers followed the general data protection regulation and informed the nurses they had the right to know what personal data existed about them (Troeth and Kucharczyj, 2018). The research process followed the Consolidated criteria for reporting qualitative research guidelines (Tong *et al*., 2007). This is in order to describe a transparent method part and to report all the necessary steps taken to strengthen the credibility of the study.

## Results

**Figure 1.**
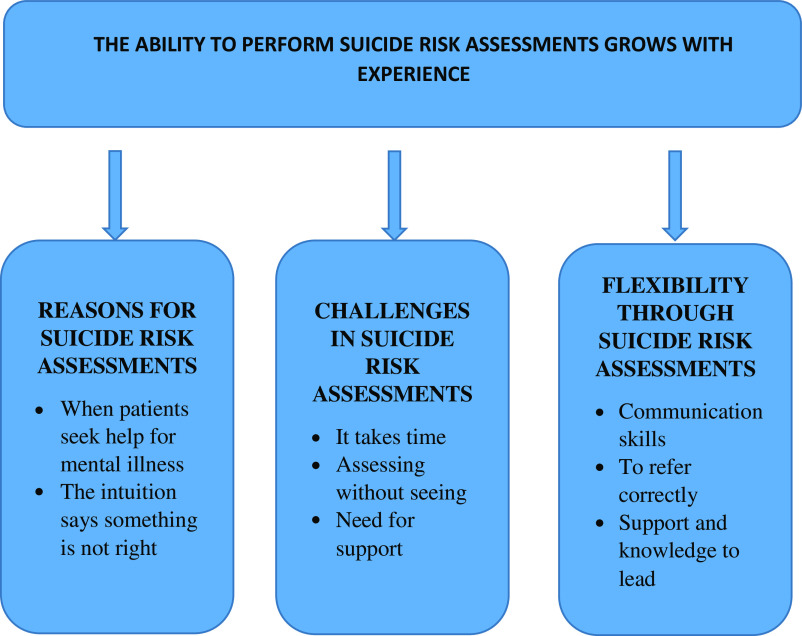
The categories and subcategories illustrating nurses’ experiences of suicide risk assessment in telephone counselling

The main result consists of four categories and their belonging subcategories. The category “The performance to do suicide risk assessments grows with experience” is hierarchically superior and illustrates how suicide assessment in TC is dependent on skills gained through experience (See Figure1).

## The performance of suicide risk assessments grows with experience

The informants said that suicide risk assessments in TC were their task and responsibility. Although there was a variation in how often they considered suicide risk assessments in TC, it was described as something done relatively frequently. The importance of having experience to perform these assessments permeated the interviews. As a junior nurse, suicide risk assessment in TC can lead to feelings of inadequacy and a feeling of lack of competence for the task. There was also uncertainty and fear about asking questions about suicidal thoughts among the inexperienced nurses. Those with more experience had greater confidence in asking questions about suicide, a confidence that had emerged through experience. All nurses described that with increased experience, the nurses’ ability to make suicide risk assessments grows and made them feel more secured.

*It’s not so strange anymore actually, because I’m used to ask those questions. Now, it’s not unpleasant or anything. In the beginning I was afraid to push them down in any way…Then you’ve learned that you save lives by asking questions (Participant (P) 15).*

## Reasons for suicide risk assessments

### When patients seek help for mental illness

The nurses said that they always asked questions about suicidal thoughts when they talked to patients who called the TC due to mental illness. Although these conversations were common, they were perceived as challenging and asking questions about suicidality was described as difficult. At the same time, an awareness was described that these questions had to be asked.

*“They may already feel down and then you might destroy their self-confidence even more by insulting them to ask - Do you want to take your own life? But at the same time, I think you save lives by asking anyway (P5).”*

By asking the patient questions about suicidal thoughts, the nurses felt that a conversation about the subject opened up. This was described by the nurses as helpful for the patient as conversations about suicide can be difficult for them to initiate. Furthermore, the nurses felt that the patients could handle questions about suicide in a good way. The nurses described how many patients with mental illness called in due to a somatic condition. It was during the conversation that it emerged that they were actually feeling mentally ill. When it is partially hidden in this way, detective work is required to ask questions that capture thoughts and feelings about suicide. A challenge in this detective work is patients who are withdrawn and quiet.

*” Then I ask a straightforward and honest question… Have you ever considered harming yourself? Then, you can open up possibilities for more such questions. I think it’s easier if I ask that question than if they themselves express it (P3).”*

### The intuition says something is not right

The nurses listened to their intuition in regard to mental illness and in the assessment of when a suicide risk assessment might be necessary. Through this intuition, the risk of suicide can be detected even in patients who have not initially discussed mental illness or suicidal thoughts. The nurses said that they needed to be sensitive to what was said between the lines as the assessment is the basis for whether the patient needs direct care or is referred further in the care system. Therefore, it is important to listen to the patient’s voice and nuances in the conversation as part of the suicide risk assessment. Several nurses referred to this intuition as a “gut feeling”; a feeling that something is not right or that a situation needs to be handled in a special way. They stated the importance of taking their “gut feeling” seriously, as it was usually correct. The nurses found this was even more important when the assessment was made by telephone.

*“It depends on how the patient acts over the phone or what feeling you get; it’s a lot about what feeling you get from the patient (P7).”*

## Challenges in suicide risk assessments

### It takes time

The nurses said that TC is limited in time to 5–7 min per call and is therefore not described as appropriate for suicide risk assessments. If the calls take longer, the next patient’s telephone call will be delayed. Making a suicide risk assessment by telephone in such a short time is described as impossible. Therefore, the nurses had to take extra time for these conversations, as important information usually did not appear directly but during the conversation. To see the telephone queue grow caused stress among the nurses. Due to time pressure, some nurses avoided asking questions about suicidal thoughts, because it involved further efforts if suicide risk was detected. Stress also made it difficult for the nurses to concentrate during the conversation and created fears about missing important information and thus making an incorrect assessment.

*“It is not many minutes you have and then you sit there and feel that it has taken a quarter of an hour and I’m behind, in the schedule. Then it’s hard not to get stressed (P2).”*

### Assess without seeing

In suicide risk assessments in TC, the nurses experienced great challenges in making an assessment without seeing the patient as several important dimensions disappeared. For example, they were unable to take into consideration the patient’s body language and facial expressions. Furthermore, it was not possible for the nurses to decide if the patient was taking care of their hygiene or was emaciated. Additional challenges were when patients had difficulty communicating or did not know the language.

*“It is body language. We communicate so much with our body language. We can see attitudes, facial expressions and other expressions. And then there is much greater security as staff if you meet the patient face to face than if you only talk over the phone in these situations (P1).”*

Safety aspects emerged in the acute telephone assessment such as fear of not being able to see what the patient was doing or where he was. Further, it was difficult for the nurses to know if the patient was telling the truth or not.

### Need for support

In the interviews, a fear and an uncertainty emerged if you have asked the right questions and made a correct assessment. Suicide risk assessments evoked emotions and stress in the nurses that made it difficult for them to let go of the patient who remained in their thoughts even after the conversation was ended. Several nurses said that they held the responsibility for the patient’s life in their hands.

*“I feel like I’m holding this patient’s life in my hands. I have to solve the problem but I can’t. I’m just a single district nurse talking with you on the phone, but I can help the patient a bit along the way (P1).”*

Support from colleagues was experienced as important for airing and raising issues both in order for the patient to receive a good assessment and also to support other nurses in their assessments. Collegial support was also found important in order to be able to refer the patient further to the right professional for an in-depth assessment. As there was a good collaboration between the varying professionals in the setting, the nurses felt safer in their assessment after advice from others. Collaboration was also seen as providing security for the patients. When talking to patients experienced as more complex or difficult to assess, the nurses felt confident when other professionals in PHC or health care staff in specialist psychiatric care took over. Thus, the nurses felt that the patient would receive the help and support they needed. They also found it easier to let go of the patient if the patient was allowed an emergency appointment with the physician.

*“You do not have to solve the problems yourself; you do it together in a team (P1).”*

In occasions where no help was available or no one took over, it caused problems for the nurses in proceeding with other tasks, as the safety of the patient could not be guaranteed.

## Flexibility through suicide risk assessments

### Communication skills

To give hope to the patient was fundamental for the nurse in the conversations. It emerged as important to be available, create security and to confirm the patient. Being a professional and asking straight and clear questions were found to open up the conversation. In the conversation, the nurses also highlighted that it was important to be there for the patient and show that you really wanted to help. It was about winning the patient’s confidence so they dared to talk about their thoughts and feelings. The importance of emphasis was illustrated, as well as how the nurses sounded in the conversation.

*“It’s about listening to the patient I have in front of me, how I can respond to him, what language is used? And then I do not mean language as in Swedish, German or English, but more in what way he uses his language to communicate…how can I reach this person and communicate? It is very difficult, it is an art (P1).”*

### To refer correctly

Suicide risk assessment in TC involves deciding who needs to make a further assessment and when. It is important to assess how acute the situation is and then refer the patient to the right professional or to the right health care institution. After the assessment, the nurse needs to be able to trust that the next professional or health care institution will take over the responsibility, in order to be able to drop the case. In cases where the nurse assesses that there is an acute risk of suicide, a doctor is always consulted.

“*Yes, but I think a bit like…my role is to mediate the next contact because I am not conversationally trained, I cannot prescribe drugs, so I will be the one who helps the patient further (P4).”*

### Support and knowledge to lead

In order to be able to conduct good conversations and suicide risk assessments, the nurses emphasised the importance of routines and support. It appeared that tools and clear guidelines for conducting suicide risk assessments by telephone at several of the health centres were lacking. There was also a need for more knowledge about mental illness and suicide. The nurses perceived that the focus on training in PHC was mostly about somatic health, and that they needed more knowledge and training about mental illness in order to be able to perform suicide risk assessments. When people with mental illness contact the TC, it is common that suicide risk assessments need to be done. Many nurses felt insecure about what questions to ask and what to do with the answer.

*“I do what feels right for me. I do not know if there are any better ways perhaps to do it. I feel that I have not received any real training in it. I also feel that we probably do things a little differently here (P6).”*

Although most of the nurses were not aware of guidelines regarding suicide risk assessments, some of them said that guidelines existed, but they had varying degrees of familiarity with them.

## Discussion

The main results of this study show that suicide risk assessments in TC are complex to carry out for new and inexperienced nurses; however with more experience, they feel safer in their assessments. The nurses demand more knowledge about suicide risk assessments. Lack of routines and time complicates suicide risk assessments. In many assessments, nurses must rely on their intuition.

Suicide risk assessments in TC are a recurring task for nurses in PHC. Thus, it is important that nurses have knowledge about how to respond to suicidal patients as they constitute the patient’s first contact (Cross *et al*., 2019; Stene and Reneflot, 2019; Berntsson *et al*., 2022). In this study, it emerged, as expected, that suicide risk assessments by TC were most often carried out if the person already had a psychiatric diagnosis. Mental illness is one of the greatest health problems in the world (WHO 2022a) and is a strong risk factor for suicide (Oexle and Rüsch, 2018). During the recent Covid-19 pandemic, it has been shown that suicidal thoughts in young adults have increased (Pirkis *et al*., 2021). There are also other mental disorders associated with increased suicidality, such as ADHD, (Quenneville *et al*., 2022) depression, substance-related disorders, psychosis (Bachmann, 2018) and repeated self-harming behaviour (Ekdahl *et al*., 2011). However, it appears that suicidal intention has been found in only 44% of clinically depressed patients and 66% of new-onset depression patients (Elzinga *et al*., 2020; Harmer *et al*., 2021). It is therefore more relevant than ever that the conditions for PHC nurses in TC are improved to create prerequisites for patient safety (Sherwood and Zomorodi, 2014). In this study, the nurses described novelty and insecurity when working as junior nurses in situations when they asked questions about suicide intention, especially when the answer was affirmative. Previous research also shows that PHC staff are unsure about their ability to interpret expressed suicidal thoughts in patients (Harmer *et al*., 2021). To secure suicide prevention in accordance with the existing global guidelines, several countries have begun work on developing suicide prevention programmes (WHO 2022a), which has been evaluated positively by the staff (Elzinga *et al*., 2020). However, they take a broader approach and do not focus specifically on TC (Solin *et al*., 2021). The results of this study indicate that there is a great need to find strategies for making suicide risk assessments in TC and to consider the challenges that PHC nurses face. In Sweden, both regions and municipalities are responsible for ensuring that education takes place in their health care services (Government Offices in Sweden 2020). This work has not been sufficient, and it has become clear that the nurses in this study do not feel that they have enough knowledge. Suicide prevention needs to be given greater focus in both education and ongoing clinical work (Wärdig *et al*., 2022). Even nursing students describe that the area is not handled sufficiently during their education (Ferguson *et al*., 2020). Research has shown that short education, such as theme days with suicidology, have shown good results (Solin *et al*., 2021). In addition, carrying out suicide risk assessments via TC was perceived as an extra challenge as they lacked the visual aspect, such as what the patient looks like, facial expressions and body language. Body language and the patient’s reactions cannot be perceived by telephone; therefore, the nurse must use experience, knowledge and critical thinking in order to be able to interpret non-verbal communication (Greenberg, 2009). The patient has different ways of expressing their symptoms and the nurse’s challenge is to be able to make correct and difficult decisions (Onubogu and Earp, 2013). In this study, the nurses highlight difficulties in assessing patients who express themselves sparingly, or where language confusion occurs. In TC, the nurse has less control over the patient, and it is complicated to assess patients with difficulties in expressing themselves verbally while also being unable to read emotional signals (Gilmore and Ward-Ciesielski, 2019). Among oncology nurses, there have been efforts to use video communication when following up patients, which has been effective for some patients (Rygg *et al*., 2021). Video calls in PHC could create conditions for better suicide risk assessments as the patient is then visible.

The nurses in this study highlighted difficulties in providing patient safety in TC as they were afraid of making incorrect assessments due to stress. Suicide risk assessments must be as reliable as possible and it is vital to prevent incorrect assessments from taking place (Sherwood and Zomorodi, 2014) and in this the nurses’ competence is central (Purc-Stephenson and Trasher, 2010). It emerged that the limited time frame for the calls was a stress factor for the nurses as other patients were waiting in a telephone queue. Despite this, they took the time they needed to listen to the patient’s story and ask questions based on it. A prerequisite for being able to actively listen to the patient and be able to make the right decision is that one does not feel stressed (Berntsson *et al*., 2022). It is also important that the nurses have a reasonable and manageable workload. However, it is common for nurses to perform tasks that are not patient-related, which can affect the work environment and workload negatively (Anskär *et al*., 2019). Another finding in this study was that conversations about mental illness usually take longer and are difficult to assess over the phone. Still, these calls are limited in time. Time pressure can mean that a high quality of care is not achieved as stress can lead to the conversation becoming unfocused and the patient being misjudged (Purc-Stephenson and Thrasher, 2010; Skogevall *et al*., 2020). Based on this, the nurses are not given the right conditions to work effectively with suicide risk assessments in TC as PHC is designed today. It is necessary to problematise the relationship between time, trust and alliance in the nurse/patient relationship. With the current way of working, perhaps nurses in PHC have an impossible task in relation to suicide risk assessments by telephone.

The results showed that the nurses feel that intuition is an aid in suicide risk assessment in TC; however at the same time, they could feel uncertain whether their intuition was correct. Intuition is a process and not just a “gut feeling” based on care experience and knowledge. Nurses should listen to their intuition and use this knowledge (Melin-Johansson *et al*., 2017; Solin *et al*., 2021). Intuition can thus be a support in decision-making and may lead to an increase in quality and safety of the assessments (Melin-Johansson *et al*., 2017). Intuition is of great importance as some types of conversation with patients are difficult to interpret; for example, when the patient calls about a somatic disorder and the nurse’s intuition leads to questions that make it clear that it is about a mental illness. Issues related to mental health and risk of suicide may need more profound assessment, especially in PHC (Hogan and Goldstein Grumet, 2016). The employer should therefore assess the beliefs, knowledge and competencies of staff in these areas and conduct regular training to raise awareness of existing guidelines around suicidality. Studies show that stigma around mental illness and suicide still exists and is an obstacle to seeking help and revealing suicidality (Kučukalić and Kučukalić, 2017; Oexle *et al*., 2020). It is important that nurses continue to use their intuition as it is so significant.

Another finding that emerged in this study was the importance of collegial support. This can, for example, mean reflecting with colleagues. Collegiate support in the workplace is both necessary and positive and has been shown to increase nurses’ personal and professional development (Sangaleti *et al*., 2017), which is also confirmed by Purc-Stephenson and Trashers (2010) who highlight that the exchange of experience between staff leads to competence development. The nurses in this study also highlight the collegial support as important as there are no clear guidelines for how suicide risk assessments in TC should be carried out. The frequent lack of suicide risk prevention policies in PHC practices is also apparent, and even when they do exist, there may be uncertainty regarding what they entail (Saini *et al*., 2010). It is therefore important to listen to the nurses’ voices to improve suicide risk assessment in TC in PHC.

### Limitations

A limitation of the study is that the study was carried out in a Swedish PHC context, therefore transferability to other contexts must be seen in the light of this. A clear description of the participants, the context of the study and the process of data are given to make it possible for any reader to decide whether the results are transferable to another context (Patton, 2015). The sample consisted only of women, which reflects the gender ratio of the total population of registered nurses in Sweden up to 2020, of whom 90% were women (National Board of Health and Welfare 2021c). Based on the broad range of variation regarding the background demographics of the participants, we would argue that our sample includes a sufficient range of participants (Polit and Beck, 2021). However, in future studies, it would be relevant to also study men’s perspectives on the same research question. In qualitative studies, content-rich data is of more value than how many interviews are conducted. The data were rich in content and during the last interviews it emerged that no new data occurred, which strengthens the trustworthiness (Patton, 2015), which can be considered as data saturation. The nurses were recruited from nine different PHC centres. Thus, the participants’ experiences were not coloured by the culture of a single setting (Polit and Beck, 2021). When conducting a content analysis, there is a risk that authors do not understand the context in full and therefore fail to identify key categories (Hsieh and Shannon, 2005). To understand the context in which this study was performed, close cooperation between the authors was established. All authors are nurses in their basic profession and have a good knowledge of what nursing at a PHC centers means, as well as experience of both mental health nursing and somatic care. In the analysis process, internal validity was ensured via peer debriefing (Hsieh and Shannon, 2005), which involved all authors agreeing on the development of categories to strengthen the trustworthiness of the results.

### Conclusion

As suicide risk assessment in TC is a common duty for nurses in PHC, they need to be listened to and given resources to prevent suicide and ensure patient safety. Based on our results, the nurses are not given right conditions to conduct suicide risk assessments in TC. It is therefore important that the nurses are offered suicide preventive interventions suitable for assessment in TC. One improvement could be to use video calls. A prerequisite for making correct assessments is that the nurses can focus and take the time needed for these conversations, and that the organisation creates conditions for this.
